# Association of physiological discordance between fractional flow reserve and non-hyperaemic pressure ratios with lipid-rich plaque burden

**DOI:** 10.1093/ehjopen/oeag155

**Published:** 2026-09-18

**Authors:** Hirohiko Aikawa, Kota Murai, Yu Kataoka, Yuriko Nakaoku, Soshiro Ogata, Kentaro Mitsui, Takamasa Iwai, Kenichiro Sawada, Hideo Matama, Hiroyuki Miura, Satoshi Honda, Masashi Fujino, Shuichi Yoneda, Kazuhiro Nakao, Kensuke Takagi, Yasuhide Asaumi, Kunihiro Nishimura, Teruo Noguchi

**Affiliations:** Department of Cardiovascular Medicine, National Cerebral and Cardiovascular Center, 6-1 Kishibe-Shimmachi, Suita, Osaka 564-8565, Japan; Senri Critical Care Medical Center, Saiseikaisenri Hospital, 1-1-6 Tsukumodai, Suita, Osaka 565-0862, Japan; Department of Cardiovascular Medicine, National Cerebral and Cardiovascular Center, 6-1 Kishibe-Shimmachi, Suita, Osaka 564-8565, Japan; Department of Cardiovascular Medicine, National Cerebral and Cardiovascular Center, 6-1 Kishibe-Shimmachi, Suita, Osaka 564-8565, Japan; Department of Preventive Medicine and Epidemiology, National Cerebral and Cardiovascular Center, 6-1 Kishibe-Shimmachi, Suita, Osaka 564-8565, Japan; Department of Preventive Medicine and Epidemiology, National Cerebral and Cardiovascular Center, 6-1 Kishibe-Shimmachi, Suita, Osaka 564-8565, Japan; Department of Cardiovascular Medicine, National Cerebral and Cardiovascular Center, 6-1 Kishibe-Shimmachi, Suita, Osaka 564-8565, Japan; Department of Cardiovascular Medicine, National Cerebral and Cardiovascular Center, 6-1 Kishibe-Shimmachi, Suita, Osaka 564-8565, Japan; Department of Cardiovascular Medicine, National Cerebral and Cardiovascular Center, 6-1 Kishibe-Shimmachi, Suita, Osaka 564-8565, Japan; Department of Cardiovascular Medicine, National Cerebral and Cardiovascular Center, 6-1 Kishibe-Shimmachi, Suita, Osaka 564-8565, Japan; Department of Cardiovascular Medicine, National Cerebral and Cardiovascular Center, 6-1 Kishibe-Shimmachi, Suita, Osaka 564-8565, Japan; Department of Cardiovascular Medicine, National Cerebral and Cardiovascular Center, 6-1 Kishibe-Shimmachi, Suita, Osaka 564-8565, Japan; Department of Cardiovascular Medicine, National Cerebral and Cardiovascular Center, 6-1 Kishibe-Shimmachi, Suita, Osaka 564-8565, Japan; Department of Cardiovascular Medicine, National Cerebral and Cardiovascular Center, 6-1 Kishibe-Shimmachi, Suita, Osaka 564-8565, Japan; Department of Cardiovascular Medicine, National Cerebral and Cardiovascular Center, 6-1 Kishibe-Shimmachi, Suita, Osaka 564-8565, Japan; Department of Cardiovascular Medicine, National Cerebral and Cardiovascular Center, 6-1 Kishibe-Shimmachi, Suita, Osaka 564-8565, Japan; Department of Cardiovascular Medicine, National Cerebral and Cardiovascular Center, 6-1 Kishibe-Shimmachi, Suita, Osaka 564-8565, Japan; Department of Preventive Medicine and Epidemiology, National Cerebral and Cardiovascular Center, 6-1 Kishibe-Shimmachi, Suita, Osaka 564-8565, Japan; Department of Cardiovascular Medicine, National Cerebral and Cardiovascular Center, 6-1 Kishibe-Shimmachi, Suita, Osaka 564-8565, Japan

**Keywords:** Fractional flow reserve, Non-hyperaemic pressure ratio, Near-infrared spectroscopy, Vulnerable plaque, Coronary artery disease

## Abstract

**Aims:**

Fractional flow reserve (FFR) and non-hyperaemic pressure ratios (NHPRs) frequently yield discordant results in clinical practice, yet the mechanisms underlying this mismatch remain uncertain. Plaque composition has been suggested to influence FFR, raising the possibility that discordance reflects the features of lipid-rich plaque (LRP). This study aimed to investigate whether physiological discordance between FFR and NHPR is associated with increased lipid burden.

**Methods and results:**

We retrospectively analysed 217 intermediate coronary lesions from 189 patients who underwent FFR, NHPR, and near-infrared spectroscopy with intravascular ultrasound imaging. Lesions were classified using established cut-offs for FFR (0.80) and NHPR (0.89). Lipid burden was quantified by the maximum lipid core burden index within any 4-mm segment (maxLCBI_4mm_). Lipid-rich plaque was defined as maxLCBI_4mm_ > 400. The clinical endpoint was 3-year target vessel failure (TVF). Physiological discordance occurred in 24.0% of lesions, predominantly as an FFR+/NHPR− pattern (19.8%). FFR was independently associated with maxLCBI_4mm_ [*β*, 65.2; 95% confidence interval (CI), 29.0–101.5; *P* < 0.001], whereas NHPR was not (*β*, 16.5; 95% CI, −15.9 to 49.0; *P* = 0.316). The FFR+/NHPR− group demonstrated the highest maxLCBI_4mm_ (525 ± 201) and the greatest prevalence of LRP (65.1%; both *P* < 0.001). In multivariable analysis, the FFR+/NHPR− pattern independently predicted LRP [adjusted odds ratio, 6.12; 95% CI, 1.20–31.28; *P* = 0.030]. TVF occurrences did not differ significantly across physiological groups.

**Conclusion:**

FFR+/NHPR− lesions exhibit markedly greater lipid burden, suggesting that hyperaemia-based indices better capture plaque vulnerability than resting physiology. This pattern may serve as a marker of plaque vulnerability, suggesting that deferral of FFR+/NHPR− lesions could warrant caution given their higher lipid burden.

## Graphical Abstract

**Graphical Abstract:**
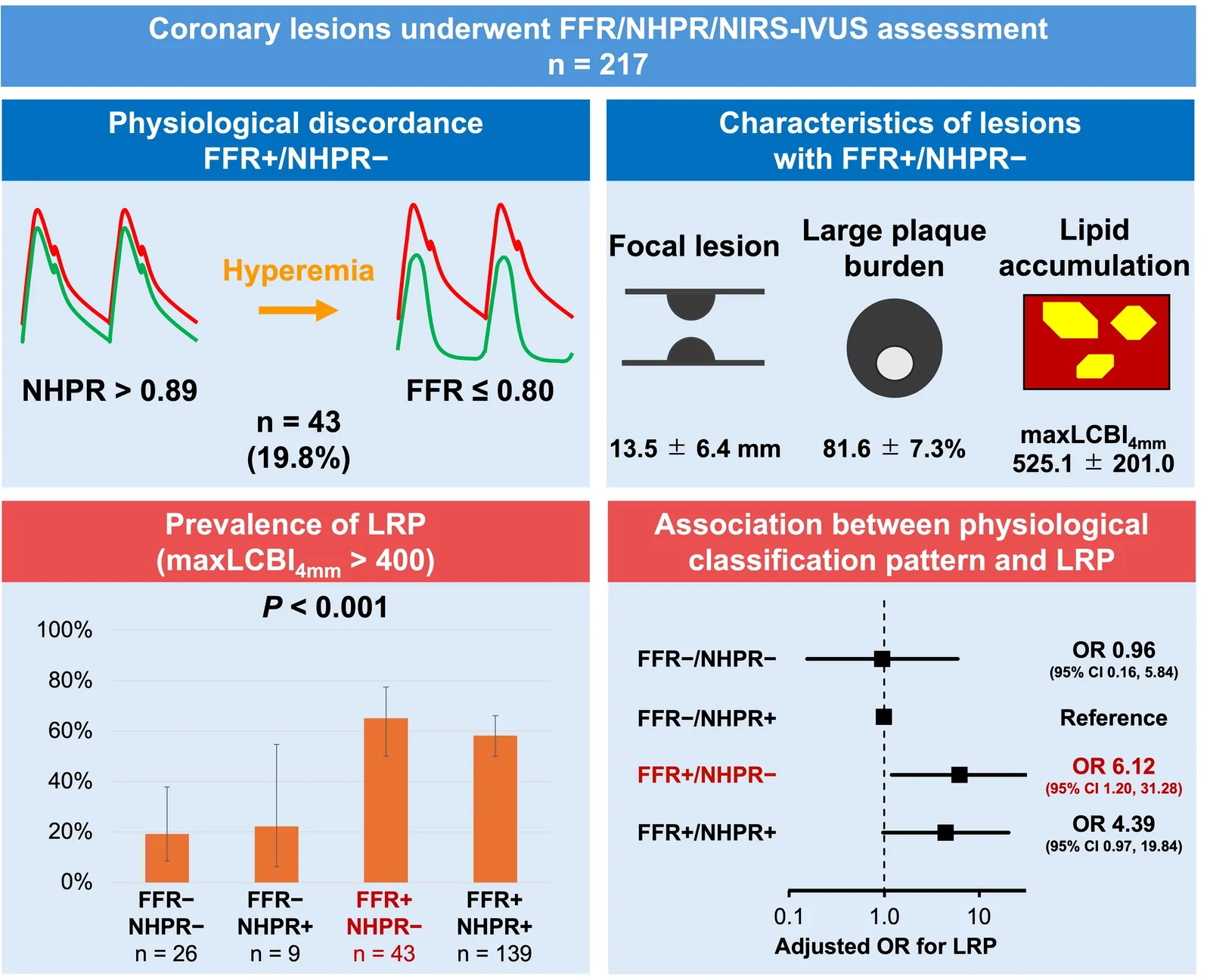

## Introduction

Coronary physiological indices, including fractional flow reserve (FFR) and non-hyperaemic pressure ratio (NHPR), are widely recommended in contemporary guidelines for the functional assessment of coronary stenosis.^1–3^ However, in clinical practice, these indices yield discordant results in ∼ 20% of lesions, which may lead to unnecessary interventions or inappropriate deferral of revascularization.^4,5^ The mechanisms underlying this mismatch between FFR and NHPR are not fully understood. Notably, FFR is measured during pharmacologically induced hyperaemia, whereas NHPRs are obtained under resting conditions. Therefore, any pathophysiological factor that alters the coronary vasodilatory response may differentially affect these indices, resulting in discordant interpretations.

Recent evidence suggests that plaque morphology may influence FFR.^6–9^ We recently employed near-infrared spectroscopy (NIRS) to quantitatively assess lipid content within coronary plaques.^10^ In that analysis, a lower distal coronary pressure-to-aortic pressure ratio (Pd/Pa) during hyperaemia (i.e. lower FFR) was associated with greater lipid accumulation within the vessel wall. Furthermore, a larger reduction in Pd/Pa from rest to hyperaemia (i.e. change in Pd/Pa) was independently associated with more pronounced lipid-rich plaque features. These findings imply that lipid-rich plaques (LRPs) may attenuate the hyperaemic response, thereby influencing FFR more strongly than NHPR. Given that plaque lipid content is also a well-established predictor of future cardiovascular events, investigating the relationship between physiological indices and plaque vulnerability may enhance risk stratification beyond haemodynamic assessment alone.

Accordingly, this study aimed to evaluate lipid core burden in lesions exhibiting discordance between FFR and NHPR using NIRS. Specifically, we focused on lesions that were FFR-positive but NHPR-negative. Clarifying the plaque characteristics associated with this discordant pattern, which is frequently encountered yet clinically ambiguous, may help improve risk assessment and guide treatment decisions in patients with coronary artery disease.

## Methods

The data that support the findings of this study are available from the corresponding author upon reasonable request.

### Study population

This retrospective study included patients with chronic coronary syndrome who underwent coronary angiography followed by physiological assessment with FFR and NHPR, as well as NIRS-intravascular ultrasound (IVUS) imaging for intermediate coronary stenosis at our institution. Intermediate stenosis was defined as a lesion with 25–75% diameter stenosis on quantitative coronary angiography (QCA).

The study protocol adhered to the ethical principles outlined in the Declaration of Helsinki (1975). The protocol was approved by the Institutional Review Board of the National Cerebral and Cardiovascular Center (M24-055-11). The subjects gave informed consent.

### Quantitative coronary angiography analyses

Coronary angiography was performed following intracoronary administration of nitroglycerin (100–300 μg). QCA was conducted using the QAngio XA system (Medis, Leiden, the Netherlands).^11^ The analysed variables included minimum lumen diameter, reference vessel diameter, diameter stenosis, and lesion length.

### Physiological assessments

The target vessel was defined as the coronary artery in which FFR/NHPR measurement was performed. The target lesion was defined as the coronary segment presenting with the most severe stenosis within the target vessel.

FFR/NHPR measurement was performed as previously described.^12^ Briefly, after cannulation of the coronary artery with a 5–7 Fr guiding catheter, a pressure-monitoring guidewire [PressureWire Certus, Aeris, or X (Abbott Vascular, Santa Clara, CA, USA); Optowire (Zeon Medical, Tokyo, Japan); or COMET (Boston Scientific, Marlborough, MA, USA)] was advanced to the most distal site of the target vessel. Pd/Pa at rest was recorded first, followed by measurement of NHPR. Depending on the type of pressure wire used, one of the following NHPRs was recorded: resting full-cycle ratio (RFR), diastolic pressure ratio (dPR), or diastolic hyperaemia-free ratio (DFR).^13^

Subsequently, hyperaemia was induced by intravenous administration of adenosine 5′-triphosphate at a rate of 180 μg/kg/ min. In patients with contraindications to the use of adenosine 5′-triphosphate, 2 mg of intracoronary nicorandil was administered instead.^14^ After hyperaemia was achieved, Pd/Pa was measured again to obtain the FFR value. The change in Pd/Pa was calculated using the following formula:^10,15^

Change in Pd/Pa = Pd/Pa at rest − Pd/Pa during hyperaemia.

Target lesions were classified into four groups according to the following cut-off values: 0.80 for FFR and 0.89 for NHPRs:

1. FFR−/NHPR− (FFR > 0.80 and NHPR > 0.89),
2. FFR−/NHPR+ (FFR > 0.80 and NHPR ≤ 0.89),
3. FFR+/NHPR− (FFR ≤ 0.80 and NHPR > 0.89),
4. FFR+/NHPR+ (FFR ≤ 0.80 and NHPR ≤ 0.89).

All procedural decisions, including the selection of the lesion or vessel for physiological assessment, the choice of pressure-monitoring guidewire, and the type of hyperaemic agent, were left to the discretion of the individual operator.

### Near-infrared spectroscopy-intravascular ultrasound imaging acquisition and analysis

In some patients, physiological assessments were performed during diagnostic coronary angiography, whereas NIRS-IVUS imaging was subsequently performed during staged elective PCI. This clinical workflow accounted for the interval between the two assessments. The entire target vessel was evaluated using NIRS combined with an intravascular ultrasound (IVUS) imaging catheter. The decision to perform NIRS-IVUS imaging was made at the discretion of the individual operator based on the perceived clinical need for detailed assessment of coronary plaque morphology and composition; no prespecified imaging criteria were applied. Following intracoronary administration of nitroglycerin (100–300 μg), an imaging catheter (Dualpro™, Infraredx, Bedford, MA, USA) was advanced to the distal end of the target vessel and automatically pulled back at a speed of 2.0 mm/s and 1800 rpm.^16^ Near-infrared spectroscopy chemogram data were acquired using the Makoto® system (Infraredx, Bedford, MA, USA), and raw IVUS data were analysed using dedicated software (QIvus®, Medis, Leiden, The Netherlands) for quantitative IVUS analysis. Both analyses were performed by experienced analysts (H.A., K.M., Y.K., and T.I.) who were blinded to patients’ clinical information.

For NIRS analysis, the lipid core burden index (LCBI) was automatically calculated as the proportion of yellow pixels to the total number of pixels within the region of interest.^17^ The maximum LCBI within any 4-mm segment (maxLCBI_4mm_) was used as a surrogate marker of lipid burden in the target lesion. A maxLCBI_4mm_ value > 400 was defined as indicative of an LRP.^18^

For IVUS analysis, cross-sectional images were analysed at 1-mm intervals. The luminal and external elastic membrane (EEM) contours were manually traced, and the plaque area was defined as the area between these two boundaries.^19^ The minimum lumen area was measured, and plaque burden was defined as the maximum value of the plaque plus media area divided by the EEM area.^19^

### Clinical outcomes

As an exploratory analysis, we evaluated the association between physiological classification patterns and clinical outcomes. The clinical outcome was the occurrence of a target vessel failure (TVF), defined as a composite of cardiac death, target vessel-related non-fatal myocardial infarction, and ischaemia-driven target vessel revascularization (ID-TVR).^5^ Cardiac death included sudden cardiac death or death due to acute myocardial infarction, heart failure, cardiogenic shock, or other cardiovascular causes.^20^ Myocardial infarction was diagnosed according to the Universal Definition.^21^ ID-TVR was defined as any revascularization of the target vessel with angiographic diameter stenosis ≥ 50% accompanied by a positive functional study or ischaemic symptoms, or diameter stenosis ≥ 70%.^22^ Patients were followed up at our institution’s outpatient clinic, by a general practitioner, or both, at intervals of 1–3 months. Evaluation for myocardial ischaemia was performed at the discretion of the treating physicians. Clinical follow-up data were collected from medical records after the NIRS-IVUS procedure. All outcome events were adjudicated by two independent physicians (H.A. and K.M.) who were blinded to the clinical characteristics of the study participants.

### Statistical analysis

Continuous variables with a normal distribution were expressed as mean ± standard deviation, while those with a non-normal distribution were presented as median (25th–75th percentiles). Categorical variables were expressed as counts and percentages. Comparisons of normally distributed continuous variables were performed using one-way analysis of variance (ANOVA) with Tukey’s *post hoc* test for multiple comparisons. For variables with non-normal distributions, the Kruskal–Wallis test was used. Categorical variables were compared using the *χ*^2^ test, and *post hoc* pairwise comparisons were conducted with Bonferroni’s correction. Correlation between continuous variables was assessed using Pearson’s correlation coefficient. To evaluate the associations between physiological indices (FFR, change in Pd/Pa, and NHPR) and maxLCBI_4mm_, multivariable linear regression analyses were performed, with each physiological index entered into a separate model. All models were adjusted for eight clinically relevant covariates known to influence the progression of LRP: age, sex, diabetes mellitus, LDL cholesterol, lesion location in the left anterior descending artery (LAD), diameter stenosis, lesion length, and plaque burden.^23–26^ We further evaluated the association between physiological lesion classifications and the presence of LRP. Lesions were categorized as FFR−/NHPR−, FFR+/NHPR−, or FFR+/NHPR+, using FFR−/NHPR+ as the reference group. A multivariable logistic regression analysis was performed using generalized estimating equations (GEEs) to account for the within-patient correlation arising from the inclusion of multiple lesions from the same patient. The same eight covariates were included for adjustment. Multicollinearity among the variables included in the multivariable GEE model was assessed using variance inflation factors (VIFs). Model adequacy was evaluated using simulation-based residual diagnostics, implemented via the DHARMa R package.^27^ Quantile–quantile (Q–Q) plots were used to assess the overall residual distribution, and plots of residuals against predicted values were used to evaluate systematic deviations in fit and heteroscedasticity.

Given the limited sample size, the stability of the effect estimates was further assessed by comparing the odds ratios (ORs) from the univariable and fully adjusted multivariable models for both the primary analysis using FFR−/NHPR+ lesions as the reference category and the sensitivity analysis using FFR−/NHPR− lesions as the reference category. Time-to-event outcomes were calculated using Kaplan–Meier estimates and compared using the log-rank test. There were no missing data for any variables included in the analyses; therefore, no imputation was performed, and no observations were excluded because of missing data. All statistical tests were two-sided. *P* < 0.05 was regarded as statistically significant. Analyses were performed using STATA version 19.0 (StataCorp LLC, College Station, TX, USA), SPSS version 27 (IBM Corporation, Armonk, NY, USA), and the statistical software R version 4.4.0 (R Core Team, R Foundation for Statistical Computing, Vienna, Austria).^28^

## Results

### Baseline clinical demographics of the study patients

Between December 2018 and January 2025, invasive physiological assessments were performed in 2756 patients. Of these, 189 patients with 217 vessels who also underwent NIRS-IVUS imaging met the eligibility criteria and were included in the present analysis (*Figure 1*). Compared with patients excluded from the analysis, the included patients were younger (69.5 ± 10.0 vs. 72.8 ± 10.1 years, *P* < 0.001) and more frequently male (81.5% vs. 75.1%, *P* = 0.048). They also had higher prevalences of hypertension, dyslipidaemia, diabetes mellitus, chronic kidney disease, and prior myocardial infarction (see Supplementary material online, *Table S1*). The time interval between FFR/NHPR measurement and NIRS-IVUS imaging was 28 (3–50) days. No cardiovascular events occurred during this interval. In patients undergoing staged PCI, antiplatelet therapy was initiated or intensified as clinically indicated, whereas no substantial changes were made to other cardiovascular medications. The distribution of FFR and NHPR values is shown in *Figure 2*. Among these vessels, physiological discordance between FFR and NHPR was observed in 52 vessels (24.0%). Specifically, FFR−/NHPR+ discordance occurred in 9 vessels (4.1%), while FFR+/NHPR− discordance was observed in 43 vessels (19.8%). The baseline clinical characteristics of the study population are summarized in *Table 1*. The mean age was 69.5 years, and 81.5% of the participants were male. The cohort exhibited a high prevalence of hypertension (83.6%), dyslipidaemia (90.5%), and type 2 diabetes mellitus (52.9%). Among the subgroups, patients with FFR+/NHPR− lesions had the lowest haemoglobin A1c levels. Regarding medication use, aspirin was more frequently prescribed in patients with FFR + lesions.

**Figure 1:**
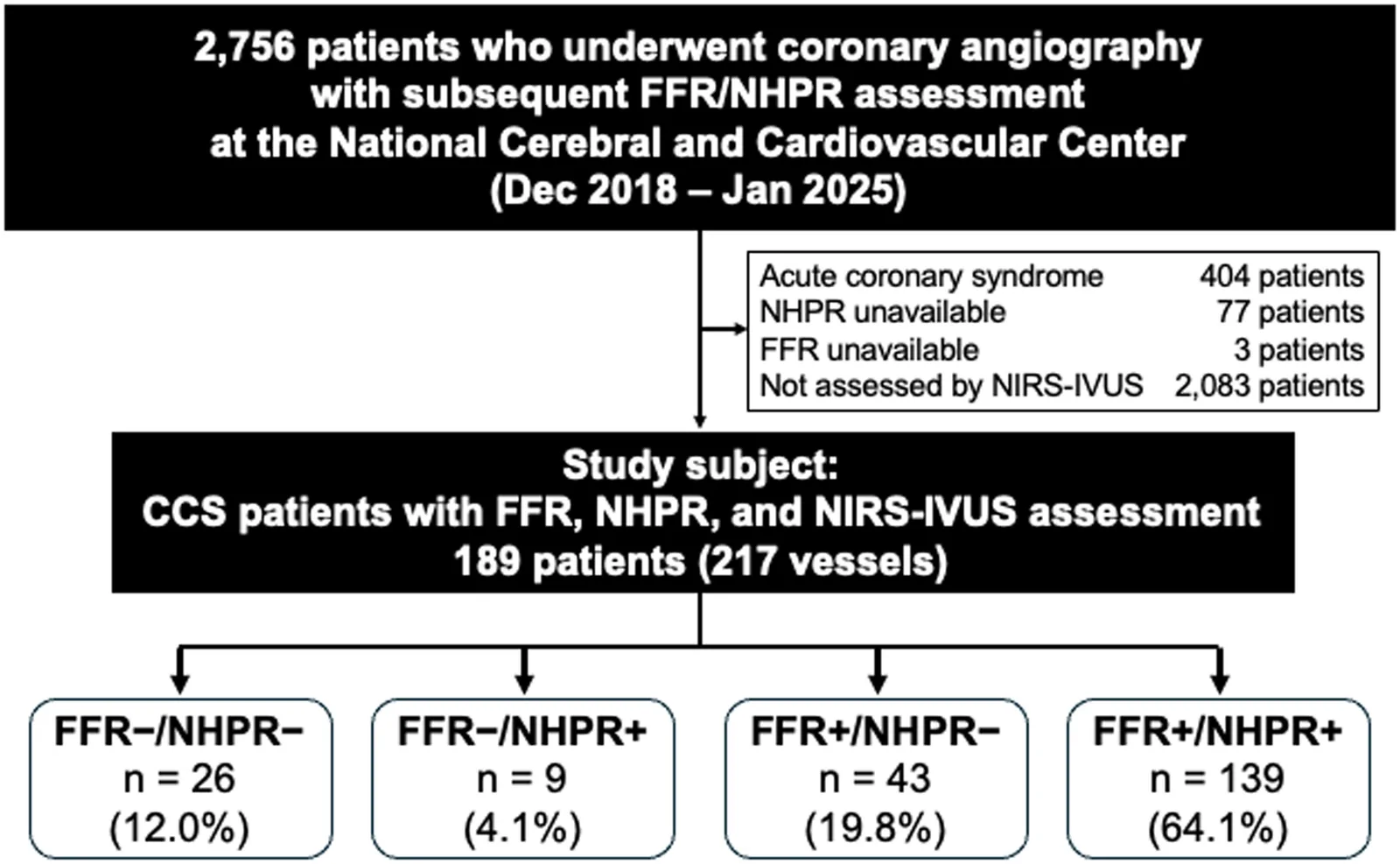
Study flowchart. A total of 189 patients with CCS, comprising 217 vessels that underwent FFR, NHPR, and NIRS-IVUS assessments, were included in the study. CCS, chronic coronary syndrome; FFR, fractional flow reserve; IVUS, intravascular ultrasound; NHPR, non-hyperaemic pressure ratio; NIRS, near-infrared spectroscopy.

**Figure 2:**
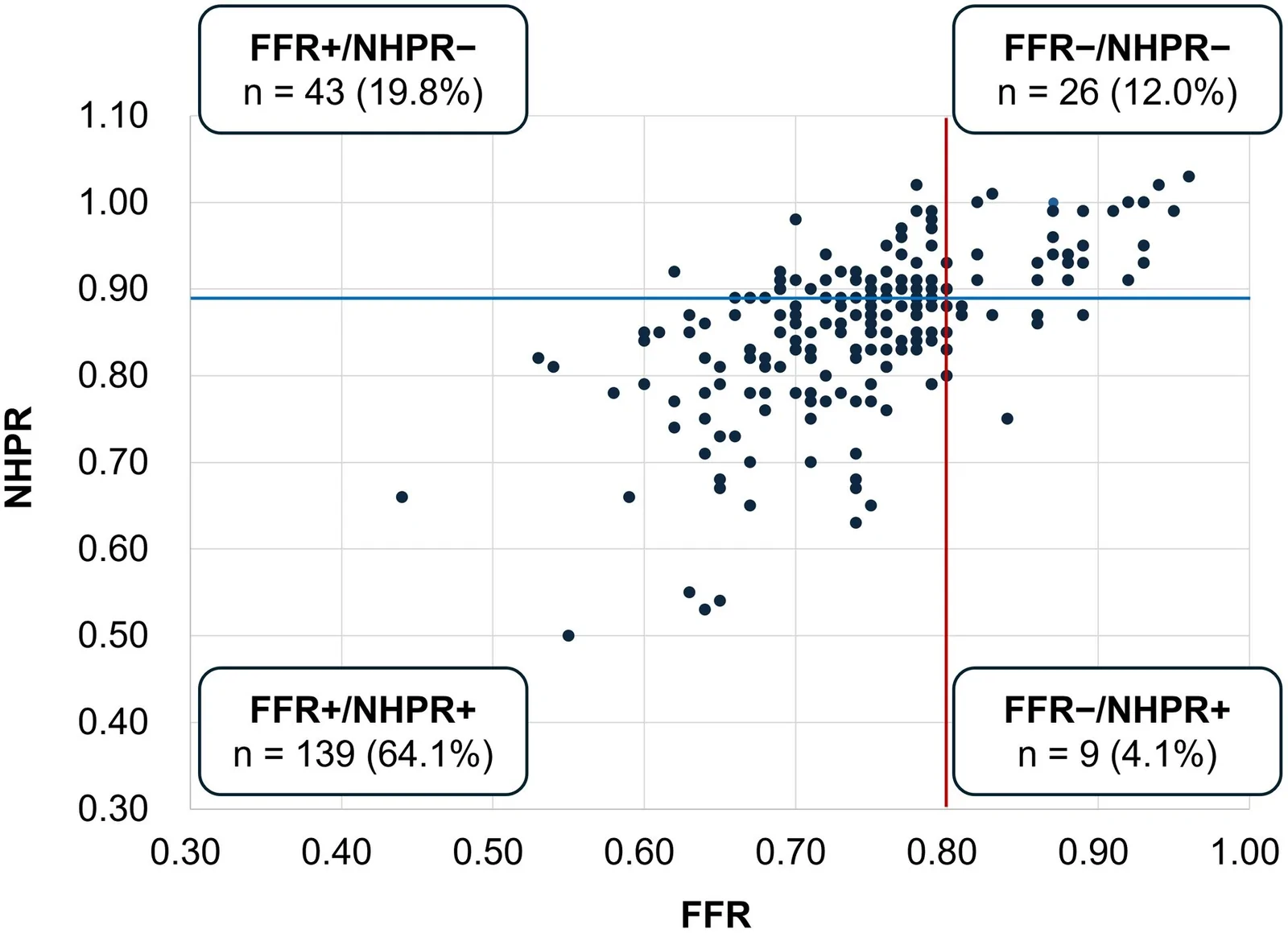
Distribution of FFR and NHPR. Physiological discordance was observed in 24.0% of lesions, with the FFR+/NHPR− pattern accounting for the majority (19.8%). The vertical and horizontal lines indicate the cut-offs for FFR (0.80) and NHPR (0.89), respectively. Abbreviations are as given in *Figure 1*.

**Table 1:** Baseline clinical characteristics by physiological classification groups

	Overall^a^ *n* = 197	FFR−/NHPR− *n* = 15	FFR−/NHPR+ *n* = 5	FFR +/NHPR− *n* = 40	FFR +/NHPR+ *n* = 129	*P* value
Age, years	69.5 ± 10.0	69.4 ± 10.7	73.4 ± 10.2	67.9 ± 10.8	69.9 ± 9.7	0.564
Male, *n* (%)	154 (81.5%)	12 (80.0%)	3 (60.0%)	31 (77.5%)	108 (83.7%)	0.494
Body mass index, kg/m^2^	24.6 ± 3.2	24.4 ± 1.5	24.1 ± 4.3	24.3 ± 3.2	24.7 ± 3.3	0.872
Hypertension, *n* (%)	158 (83.6%)	14 (93.3%)	4 (80.0%)	31 (77.5%)	109 (84.5%)	0.523
Dyslipidaemia, *n* (%)	171 (90.5%)	13 (86.7%)	5 (100%)	36 (90.0%)	117 (90.7%)	0.850
Diabetes mellitus, *n* (%)	100 (52.9%)	11 (73.3%)	3 (60.0%)	18 (45.0%)	68 (52.7%)	0.306
Current smoking, *n* (%)	25 (13.2%)	2 (13.3%)	0 (0.0%)	7 (17.5%)	16 (12.4%)	0.688
Prior myocardial infarction, *n* (%)	58 (30.7%)	8 (53.3%)	1 (20.0%)	15 (37.5%)	34 (26.4%)	0.117
Haemodialysis, *n* (%)	12 (6.3%)	1 (6.7%)	0 (0.0%)	2 (5.0%)	9 (7.0%)	0.908
LVEF, %	60 (55**–**65)	60 (59**–**65)	60 (58**–**65)	60 (50**–**64)	60 (53**–**65)	0.451
Laboratory data						
T-Chol, mg/dL	146.3 ± 30.4	141.3 ± 22.4	133.2 ± 23.3	145.9 ± 33.8	147.6 ± 30.4	0.669
LDL-C, mg/dL	72.9 ± 22.7	69.5 ± 18.8	57.0 ± 23.3	72.1 ± 22.1	74.2 ± 23.2	0.353
HDL-C, mg/dL	48.3 ± 14.3	47.6 ± 14.3	56.6 ± 10.3	47.8 ± 9.0	48.3 ± 15.8	0.859
Triglyceride, mg/dL	128 (95**–**183)	133 (127**–**184)	136 (62**–**200)	116 (88**–**155)	127 (95**–**195)	0.302
Haemoglobin A1c, %	6.5 ± 0.9	7.1 ± 1.0	6.8 ± 0.8	6.3 ± 0.7	6.5 ± 0.9	0.016
eGFR, mL/min per 1.73 m^2^	60.5 ± 20.4	63.0 ± 11.8	73.0 ± 15.2	61.5 ± 18.4	59.4 ± 21.8	0.460
Medication						
Aspirin, *n* (%)	180 (95.2%)	12 (80.0%)	3 (60.0%)	39 (97.5%)	126 (95.2%)	<0.001
P2Y12 inhibitor, *n* (%)	186 (98.4%)	14 (93.3%)	5 (100%)	39 (97.5%)	128 (98.4%)	0.345
Statin, *n* (%)	181 (95.8%)	15 (100%)	5 (100%)	40 (100%)	121 (93.8%)	0.274
Beta-blocker, *n* (%)	103 (54.5%)	10 (66.7%)	3 (60.0%)	19 (47.5%)	71 (55.0%)	0.623
ACE inhibitor/ARB, *n* (%)	124 (65.6%)	12 (80.0%)	2 (40.0%)	29 (72.5%)	81 (62.8%)	0.248
Insulin, *n* (%)	27 (14.3%)	2 (13.3%)	2 (40.0%)	3 (7.5%)	20 (15.5%)	0.224

^a^Patients with multiple lesions were assigned based on the lesion with the lowest FFR.

ACE, angiotensin converting enzyme; ARB, aldosterone receptor blocker; eGFR, estimated glomerular filtration rate; FFR, fractional flow reserve; HDL-C, high-density lipoprotein cholesterol; LDL-C, LDL cholesterol; LVEF, left ventricular ejection fraction; NHPR, non-hyperaemic pressure ratio; T-Chol, total cholesterol.

### Lesion characteristics and treatment strategies

Lesion-level anatomical and physiological characteristics, along with treatment strategies, are summarized in *Table 2*. Seventy per cent of the target lesions were located in the LAD. According to the QCA analysis, the FFR+/NHPR− group had the shortest lesion length among the four groups. The mean FFR and NHPR values were 0.84 and 0.86, respectively, in the FFR−/NHPR+ group, and 0.75 and 0.93, respectively, in the FFR+/NHPR− group (*Table 2*). Intravascular ultrasound analysis revealed that the greatest plaque burden was observed in the FFR+/NHPR− group. Revascularization was deferred in the majority of lesions in the FFR−/NHPR− group and the FFR−/NHPR+ group (100% and 77.8%, respectively). In contrast, nearly all lesions in the FFR+/NHPR− and FFR+/NHPR+ groups underwent revascularization (95.3% and 97.8%, respectively).

**Table 2:** Lesion characteristics, physiological measurements, and treatment strategies across physiological classification groups

	Overall *n* = 217	FFR−/NHPR− *n* = 26	FFR−/NHPR+ *n* = 9	FFR+/NHPR− *n* = 43	FFR+/NHPR+ *n* = 139	*P* value
Location of the target lesion						0.006
LMT, *n* (%)	6 (4.3%)	1 (3.8%)	0 (0.0%)	0 (0.0%)	6 (4.3%)	
LAD, *n* (%)	152 (70.0%)	10 (38.5%)	7 (77.8%)	27 (62.8%)	108 (77.7%)	
LCX, *n* (%)	22 (10.2)	5 (19.2%)	1 (11.1%)	6 (14.0%)	10 (7.2%)	
RCA, *n* (%)	36 (16.6%)	10 (38.5%)	1 (11.1%)	10 (23.3%)	15 (10.8%)	
QCA analysis						
In-stent restenosis lesion, *n* (%)	7 (3.2%)	1 (3.8%)	2 (22.2%)	1 (2.3%)	3 (2.2%)	<0.011
Minimum lumen diameter, mm	1.27 ± 0.43	1.58 ± 0.48	1.52 ± 0.32	1.33 ± 0.40	1.18 ± 0.40	<0.001
Reference lumen diameter, mm	2.68 ± 0.65	2.74 ± 0.63	3.10 ± 0.46	2.78 ± 0.80	2.60 ± 0.60	0.082
Diameter stenosis, %	52.7 ± 12.0	47.6 ± 11.9	50.2 ± 10.4	51.0 ± 12.9	54.3 ± 11.6	0.038
Lesion length, mm	18.5 ± 12.2	15.4 ± 9.6	21.5 ± 9.4	13.5 ± 6.4	20.5 ± 13.5	0.004
Physiological analysis						
Pd/Pa at rest	0.90 ± 0.06	0.98 ± 0.03	0.91 ± 0.03	0.94 ± 0.04	0.88 ± 0.05	<0.001
FFR	0.75 ± 0.08	0.89 ± 0.04	0.84 ± 0.03	0.75 ± 0.04	0.71 ± 0.06	<0.001
Change in Pd/Pa	0.16 ± 0.06	0.10 ± 0.04	0.08 ± 0.03	0.18 ± 0.05	0.16 ± 0.06	<0.001
NHPR	0.86 ± 0.09	0.96 ± 0.04	0.86 ± 0.04	0.93 ± 0.03	0.82 ± 0.08	<0.001
NHPR type						0.880
RFR	63 (29.0%)	7 (26.9%)	3 (33.3%)	13 (30.2%)	40 (28.8%)	
dPR	147 (67.7%)	18 (69.2%)	6 (66.7%)	30 (69.8%)	93 (66.9%)	
DFR	7 (3.2%)	1 (3.8%)	0 (0.0%)	0 (0.0%)	6 (4.3%)	
IVUS analysis						
Minimum lumen area, mm^2^	2.75 ± 1.70	5.11 ± 3.02	3.00 ± 1.34	2.61 ± 1.47	2.34 ± 0.93	<0.001
Plaque burden, %	79.8 ± 7.5	74.3 ± 9.7	78.7 ± 6.5	81.6 ± 7.3	80.3 ± 6.8	<0.001
Treatment strategy						<0.001
Revascularization	179 (82.5%)	0 (0.0%)	2 (22.2%)	41 (95.3%)	136 (97.8%)	
Medication	38 (17.5%)	26 (100%)	7 (77.8%)	2 (4.7%)	3 (2.2%)	

DFR, diastolic hyperaemia-free ratio; dPR, diastolic pressure ratio; IVUS, intravascular ultrasound; LAD, left anterior descending artery; LCX, left circumflex artery; LMT, left main trunk; Pd/Pa, distal coronary pressure-to-aortic pressure ratio; RCA, right coronary artery; RFR, resting full-cycle ratio; other abbreviations are as given in *Table 1*.

### Association of the physiological features and the extent of lipid burden

According to the NIRS analysis, FFR was significantly correlated with maxLCBI_4mm_ (*r* = −0.319, *P* < 0.001) (*Figure 3A*). A significant correlation was also observed between the change in Pd/Pa and maxLCBI_4mm_ (*r* = 0.290, *P* < 0.001) (*Figure 3B*). In contrast, no significant correlation was found between NHPR and maxLCBI_4mm_ (*r* = −0.130, *P* = 0.056) (*Figure 3C*). Even after adjustment for clinically relevant covariates, FFR remained independently associated with maxLCBI_4mm_ [per 0.1-unit decrease in FFR: *β* = 65.2; 95% confidence interval (CI), 29.0–101.5; *P* < 0.001] (*Figure 3D*). Similarly, the change in Pd/Pa also demonstrated a significant independent association (per 0.1-unit increase: *β* = 93.3; 95% CI, 46.4–140.2; *P* < 0.001), with a numerically higher regression coefficient than that of FFR. However, NHPR still showed no significant association in the multivariable analysis (per 0.1-unit decrease: *β* = 16.5; 95% CI, −15.9 to 49.0; *P* = 0.316).

**Figure 3:**
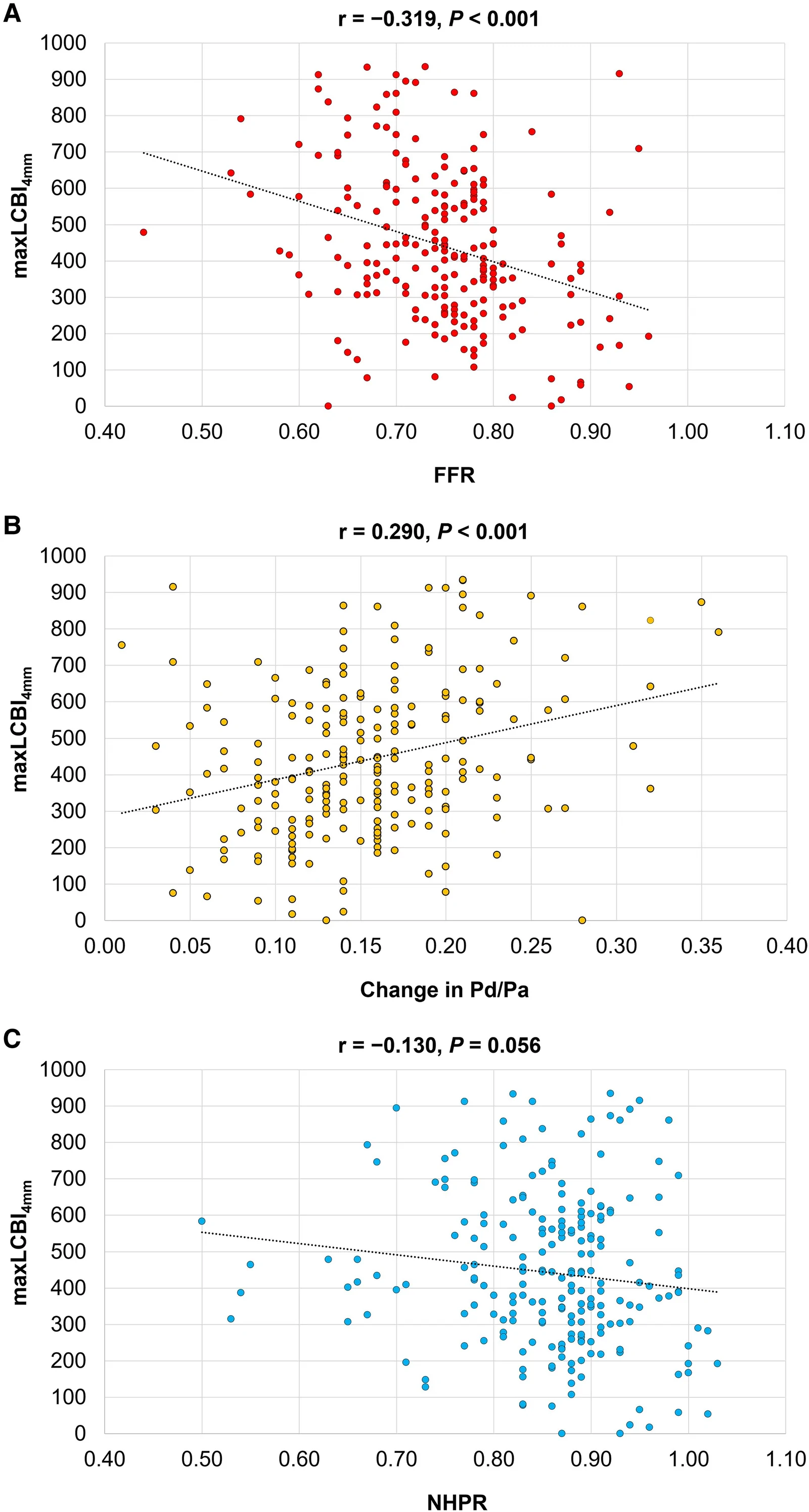

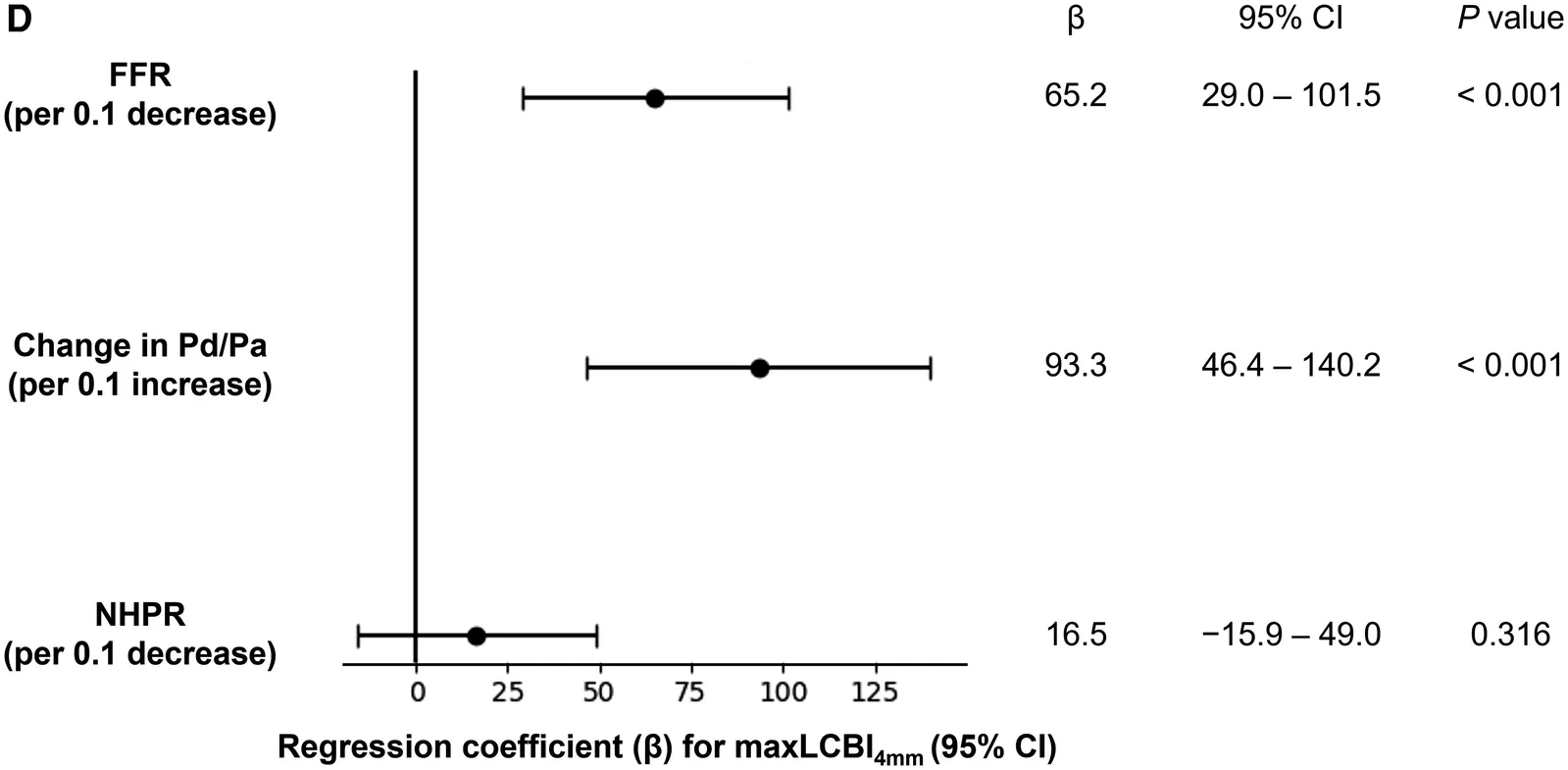
Correlations and multivariable linear regression analyses of the associations between physiological indices and maxLCBI_4mm_. (*A*) FFR and maxLCBI_4mm_. (*B*) Change in Pd/Pa and maxLCBI_4mm_. (*C*) NHPR and maxLCBI_4mm_. (*D*) Multivariable linear regression analyses for maxLCBI_4mm_. There was a significant correlation of FFR and change in Pd/Pa with maxLCBI_4mm_, whereas NHPR showed no significant association. In multivariable linear regression analyses, FFR and change in Pd/Pa were independently associated with maxLCBI_4mm_, whereas NHPR was not. MaxLCBI_4mm_, maximum lipid core burden index within any 4-mm segment; Pd/Pa, distal coronary pressure–aortic pressure ratio; CI, confidence interval; other abbreviations are as given in *Figure 1*.

FFR-positive lesions demonstrated significantly higher max LCBI_4mm_ values compared with FFR-negative lesions (469.5 vs. 303.1, *P* < 0.001). However, there was no significant difference in maxLCBI_4mm_ between NHPR-positive and NHPR-negative lesions (446.8 vs. 433.9, *P* = 0.679). When target lesions were stratified into four groups based on physiological classification, FFR+/NHPR− lesions showed the highest maxLCBI_4mm_ values among all groups (525.1 ± 201.0, *P* < 0.001) (*Figure 4A*). Similarly, the prevalence of LRP was significantly higher in FFR-positive lesions than in FFR-negative lesions (59.9% vs. 20.0%, *P* < 0.001), whereas no significant difference was observed between NHPR-positive and NHPR-negative lesions (56.1% vs. 47.8%, *P* = 0.256). Among the four physiological groups, FFR+/NHPR− lesions had the highest prevalence of LRPs (65.1%, *P* < 0.001) (*Figure 4B*).

**Figure 4:**
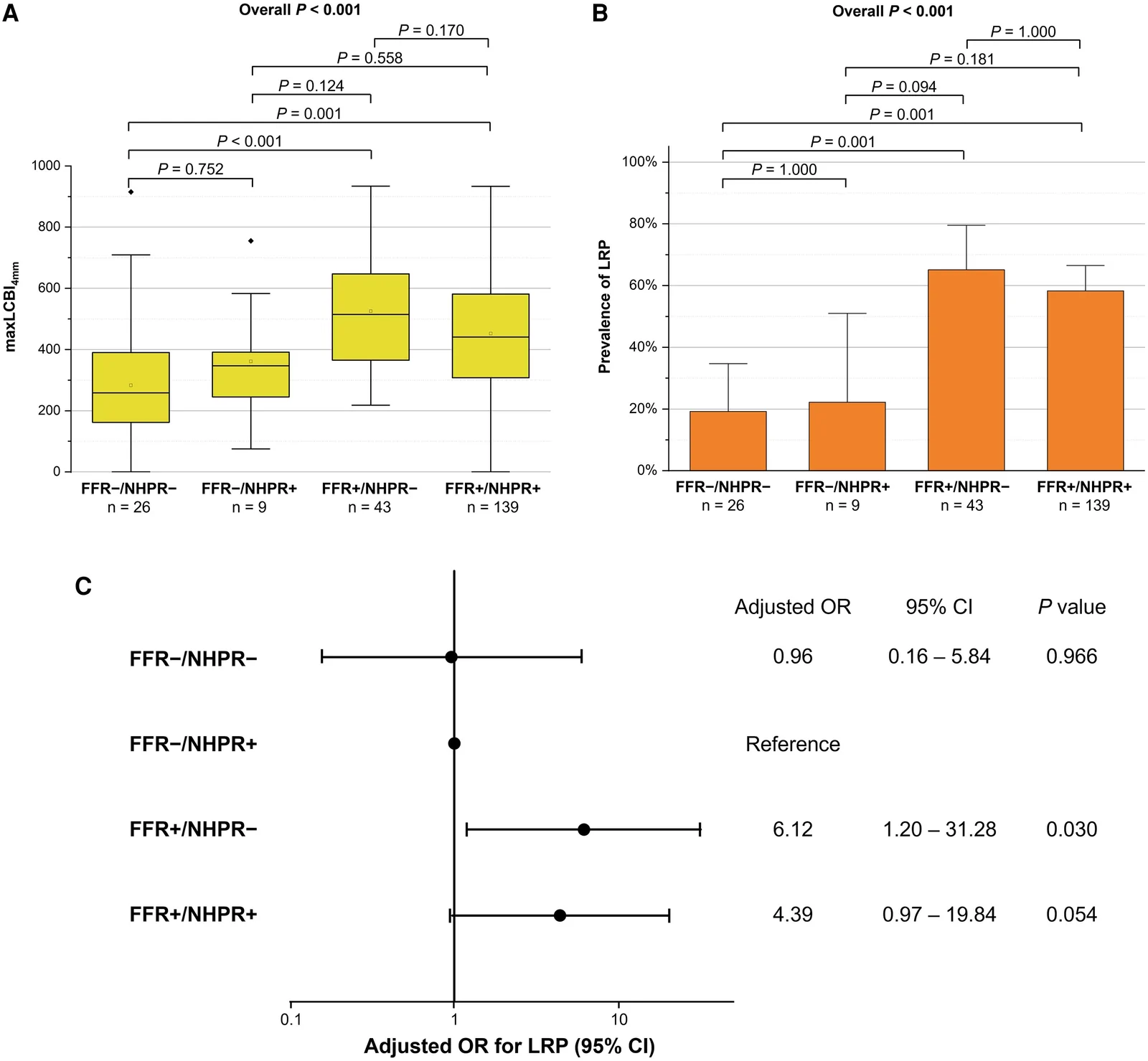
NIRS-derived lipid burden across the physiological classification groups. (*A*) MaxLCBI_4mm_. (*B*) Prevalence of LRP. (*C*) Multivariable generalized estimating equation analysis for the association between physiological classification patterns and LRP. The FFR+/NHPR− pattern demonstrated the highest maxLCBI_4mm_ and the greatest prevalence of LRP. In multivariable generalized estimating equation analyses, the FFR+/NHPR− pattern was independently associated with the presence of LRP. In panel A, the boxes indicate the interquartile range, with the mid-line representing the median. The whiskers extend from each box to the farthest point within ± 1.5 times the interquartile range. Outliers are shown as individual points. In panel B, error bars indicate 95% confidence intervals. LRP, lipid-rich plaque; OR, odds ratio; other abbreviations are as in *Figures 1* and *3*.

To further investigate the association between physiological classification and the presence of LRP, a multivariable logistic regression analysis using generalized estimating equations was performed (*Figure 4C*). Using FFR−/NHPR+ lesions as the reference group, the FFR+/NHPR− pattern was identified as an independent and significant predictor of LRP (adjusted OR, 6.12; 95% CI, 1.20–31.28; *P* = 0.030). In a sensitivity analysis using FFR−/NHPR− lesions as the reference category, the FFR+/NHPR− pattern remained independently associated with LRP (adjusted OR, 6.36; 95% CI, 1.83–22.15; *P* = 0.004; Supplementary material online, *Figure S1*), whereas the FFR−/NHPR+ pattern was not (adjusted OR, 1.04; 95% CI, 0.17–6.34; *P* = 0.966). When stratified by NHPR modality, the FFR+/NHPR− group showed a greater maxLCBI_4mm_ and a higher prevalence of LRP in both the RFR and dPR subgroups, consistent with the primary analysis (see Supplementary material online, *Table S2*). The DFR subgroup was too small for meaningful interpretation. No evidence of problematic multicollinearity was identified based on the VIFs (see Supplementary material online, *Table S3*). Simulation-based residual diagnostics showed no significant deviation from the expected residual distribution, no evidence of overdispersion or underdispersion, and no significant outliers (see Supplementary material online, *Figure S2*). The ORs were generally consistent between the univariable and fully adjusted multivariable models in both the primary and sensitivity analyses, indicating that the effect estimates were not substantially altered by covariate adjustment (see Supplementary material online, *Table S4A* and *S4B*).

### Impact of physiological classification on clinical events

During the observational period, with a median follow-up duration of 1151 days (794–1866 days), the 3-year incidence of TVF was 11.1% (see Supplementary material online, *Table S5*). TVF occurred in 17.0% of deferred lesions (*n* = 38) and 10.0% of revascularized lesions (*n* = 179).

When lesions were stratified into four physiological classification groups, the 3-year incidence of TVF did not significantly differ among the groups (log-rank *P* = 0.508) (see Supplementary material online, *Figure S3A*). In subgroup analyses according to treatment strategy, the incidence of TVF also did not significantly differ among the four physiological groups in deferred lesions (*P* = 0.930) or in revascularized lesions (*P* = 0.496) (see Supplementary material online, *Figure S3B* and *C*).

## Discussion

In this study, discordance between FFR and NHPR was observed in 24.0% of the lesions, consistent with previous studies. Among these, FFR+/NHPR− discordance accounted for ∼ 20% of all cases, indicating that it is a relatively frequent mismatch pattern encountered in clinical practice. Our findings demonstrated that FFR was significantly associated with greater lipid burden as assessed by NIRS, whereas NHPR was not. Lesions classified as FFR+/NHPR− were frequently characterized by colocalization with LRP. These observations suggest that the extent of lipid accumulation may influence physiological indices, and that the impact of plaque characteristics may differ depending on whether the index is obtained under hyperaemic or resting conditions.

This interaction may be explained by the current finding that a greater change in Pd/Pa, which reflects the vascular response to hyperaemia, was significantly associated with increased lipid burden. The mechanism underlying the observed association between hyperaemia-based physiological indices and lipid burden remains uncertain. One possible explanation is that lipid-rich plaques may exhibit an impaired vasodilatory response during pharmacological hyperaemia. Lipid accumulation is associated with inflammation, oxidative stress, and endothelial dysfunction, and previous studies have reported that necrotic or lipid-rich plaque characteristics are associated with impaired coronary endothelial function.^29–32^ Because hyperaemic agents may also induce vasodilation of the epicardial coronary arteries, an attenuated epicardial vasodilatory response could theoretically result in a relatively greater pressure gradient across lipid-rich lesions during hyperaemia.^33^ This impaired vasodilatory response potentially contributes to the observed reduction in Pd/Pa during hyperaemia in lipid-rich lesions, suggesting a possible link with endothelial dysfunction and plaque vulnerability. However, vasomotor function was not directly assessed in the present study; therefore, this proposed mechanism should be regarded as hypothesis-generating.

Previous studies have suggested that hyperaemia-based physiological indices are more closely associated with adverse plaque characteristics than resting indices. Lee et al. evaluated 214 vessels using CCTA and demonstrated that FFR was independently associated with both quantitative and qualitative high-risk plaque characteristics, whereas iFR was associated primarily with quantitative plaque features.^34^ More recently, Yang et al. reported that, among functionally significant lesions, lesions with lower FFR were independently associated with OCT-defined vulnerable plaque, including plaque rupture or thin-cap fibroatheroma.^35^ However, previous studies predominantly evaluated categorical morphological features derived from CCTA or OCT. In contrast, the present study used NIRS-IVUS, which enables direct intracoronary assessment and objective quantification of lipid burden.^17,36^ Thus, the present study provides quantitative intravascular imaging evidence supporting a specific association between FFR-positive/NHPR-negative discordance and increased coronary lipid burden.

Beyond plaque composition, lesion morphology may also contribute to physiological discordance. In addition to lipid content, we observed that FFR+/NHPR− lesions are shorter and have a larger plaque burden. Prior studies have similarly demonstrated that lesions with a large pullback pressure gradient, often characterized by focal morphology, are more likely to exhibit FFR+/NHPR− discordance.^37,38^ Furthermore, such focal lesions have been shown to harbour vulnerable plaques with higher inflammatory activity and greater necrotic content.^24,35^ Mechanistically, focal stenosis causes disturbances in laminar flow and produces low wall shear stress, thereby leading to plaque progression and inflammation.^39,40^ These findings are consistent with our observations, suggesting that both the anatomical pattern and plaque composition contribute to the physiological mismatch.

The exploratory outcome analysis showed no statistically significant difference in TVF across the physiological classification groups. However, this finding should be interpreted with considerable caution because treatment strategies differed markedly among the groups. Nearly all FFR+/NHPR− lesions underwent revascularization, whereas most FFR−/NHPR− lesions were managed with deferral. Accordingly, the present analysis does not allow an unbiased comparison of the natural prognosis associated with each physiological pattern. In this context, recent studies have highlighted the prognostic significance of FFR+/NHPR− lesions. Kuramitsu *et al*.^5^ reported a higher incidence of adverse events in deferred lesions with FFR-NHPR discordance and showed that revascularization was associated with better outcomes in the FFR+/NHPR− group compared with medical therapy. Similarly, a meta-analysis by Ha *et al*.^4^ demonstrated a reduction in cardiovascular events following revascularization in FFR+/NHPR− lesions, whereas no such benefit was observed in the FFR−/NHPR+ group. The greater lipid burden observed in our study may provide a mechanistic explanation for these clinical findings, given that non-culprit lipid-rich plaques identified by NIRS have been shown to predict future cardiovascular events, and that preventive intervention for these lesions may offer clinical benefit.^18,41,42^ These findings raise concern that deferring revascularization based solely on NHPR may leave vulnerable plaques untreated and potentially at higher risk of future events.

Recent studies have demonstrated that cardiovascular events can still occur in lesions deferred from revascularization based on physiological indices.^43,44^ As a result, clinical trials incorporating plaque characteristics evaluated by intravascular imaging have been conducted to improve risk stratification. These studies have shown that lesions with vulnerable plaque features, even when deemed physiologically non-significant, are associated with worse outcomes.^45,46^ However, in routine clinical practice, the combined use of pressure wire-based physiology and intracoronary imaging is often limited due to cost, time, and procedural complexity. In our study, two-thirds of FFR+/NHPR− lesions were found to harbour LRP, suggesting that this physiological discordance pattern may serve as a marker of plaque vulnerability. The integration of physiological discordance patterns and the change in Pd/Pa may offer a practical and less invasive approach to estimating plaque composition without relying on intravascular imaging. Such an approach may serve as a cost-effective surrogate for imaging-based risk stratification, especially in settings where intracoronary imaging is not routinely available. To validate this concept, a dedicated prospective clinical study is warranted to assess the ability of FFR, NHPR, and the change in Pd/Pa to more precisely stratify cardiovascular risk.

### Limitations

Several caveats should be considered when interpreting the current findings. First, this was a single-centre, retrospective observational study with a relatively small sample size and a limited number of cardiovascular events, which may have reduced the statistical power of the outcome analyses. Second, treatment strategies differed substantially across the physiological groups and were determined at the discretion of individual operators. In particular, nearly all FFR+/NHPR− lesions underwent revascularization, whereas most FFR−/NHPR− lesions were deferred. Therefore, the exploratory outcome analyses were strongly confounded by treatment selection, and the absence of a significant difference in TVF should not be interpreted as evidence of similar prognosis among the physiological groups. Third, NIRS-IVUS imaging was available in fewer than 10% of patients who underwent invasive physiological assessment, resulting in substantial potential for selection bias. The included patients were younger but had a greater prevalence of several cardiovascular risk factors and comorbidities than the excluded patients. Although the specific indications for NIRS-IVUS imaging were not systematically recorded, these differences suggest that NIRS-IVUS may have been preferentially used when operators suspected a greater atherosclerotic burden or considered detailed plaque characterization clinically relevant. Accordingly, the present findings may not be generalizable to an unselected population undergoing invasive physiological assessment. Fourth, although three types of NHPR (RFR, dPR, and DFR) were used, we did not evaluate their individual effects, and subtle differences between these modalities may have affected the results. Fifth, the correlation coefficients (*r*-values) between FFR or the change in Pd/Pa and maxLCBI_4mm_ were modest. Sixth, the study was conducted at a single centre in Japan, limiting the generalizability of the findings to other populations.

## Conclusion

Hyperaemia-based FFR, but not resting NHPR, was influenced by the accumulation of lipid material as assessed by NIRS. Consequently, lesions exhibiting an FFR+/NHPR− physiological pattern were characterized by a significantly greater lipid burden. These results suggest that physiological discordance may reflect underlying plaque vulnerability. Incorporating physiological discordance patterns with dynamic haemodynamic parameters may therefore improve risk stratification. Further prospective studies are warranted to validate this approach and clarify its clinical utility in the management of coronary artery disease.

## Supplementary Material

oeag155_Supplementary_Data

## Data Availability

The data underlying this article are available from the corresponding author upon reasonable request, subject to ethical approval and data sharing agreements.

## Lead author biography

Hirohiko Aikawa, MD, PhD, is a Japanese cardiologist specializing in cardiovascular emergencies and acute pericarditis. He is a staff physician at Senri Emergency and Critical Care Center, Osaka Prefectural Saiseikai Senri Hospital. He received his PhD from Kumamoto University. He received the JACC: Asia Young Author Achievement Award in 2024. Member of the Japanese Circulation Society and the Japanese Association for Acute Medicine.
