# Supplementary material for: Association of physiological discordance between fractional flow reserve and non-hyperaemic pressure ratios with lipid-rich plaque burden
Source: Eur Heart J Open. 2026 Sep 18;6(5):oeag155. doi: 10.1093/ehjopen/oeag155 (PMC13623228; doi:10.1093/ehjopen/oeag155)
Supplement: Supplementary file 1 [file oeag155_supplementary_data.pdf]

Supplementary Appendix

Association of Physiological Discordance Between Fractional Flow Reserve and Nonhyperemic Pressure Ratios With Lipid-Rich Plaque Burden

Hirohiko Aikawa, MD, PhD[a, b]; Kota Murai, MD, PhD[a]; Yu Kataoka, MD, PhD[a]; Yuriko Nakaoku, MD, PhD[c]; Soshiro Ogata, RN, PhD[c]; Kentaro Mitsui, MD[a]; Takamasa Iwai, MD, PhD[a]; Kenichiro Sawada, MD, PhD[a]; Hideo Matama, MD[a]; Hiroyuki Miura, MD, PhD[a]; Satoshi Honda, MD, PhD[a]; Masashi Fujino, MD, PhD[a], Shuichi Yoneda, MD, PhD[a];Kazuhiro Nakao, MD, PhD[a]; Kensuke Takagi, MD, PhD[a]; Yasuhide Asaumi, MD, PhD[a]; Kunihiro Nishimura, MD, PhD, MPH[c]; Teruo Noguchi, MD, PhD[a]

a Department of Cardiovascular Medicine, National Cerebral and Cardiovascular Center, Suita, Japan
b Senri Critical Care Medical Center, Saiseikaisenri Hospital, 1-1-6, Tsukumodai, Suita, Osaka 565-0862, Japan.
c Department of Preventive Medicine and Epidemiology, National Cerebral and Cardiovascular Center, Suita, Osaka, Japan

Table of Contents

Supplementary Figures

Figure S1. Multivariable Generalized Estimating Equation Analysis for the Association Between Physiological Classification Patterns and LRP, with FFR−/NHPR− as the Reference Group…………………………………………...3
Figure S2. Model Diagnostics for the Multivariable Generalized Estimating Equation Model for the Presence of LRP
(A) QQ plot residuals……………………………………………………………....4
(B) DHARMa residual vs predicted………………………………………..............5
Figure S3. Cumulative incidence of TVF across the physiological classification groups.
(A) All lesions…………………………………………………………..................6
(B) Deferred lesions……………………………………………….....................6

(C) Revascularized lesions……………………………………………………….....7

2. Supplementary Tables

Table S1. Comparison of Baseline Clinical Characteristics Between Patients Included and Excluded from the Study…………………………………………….....8
Table S2. Association Between Physiological Classification Pattern and maxLCBI4mm and LRP, Stratified by NHPR Modality…………..…………………9
Table S3. VIF for the Multivariable Model of LRP……….................................10
Table S4. Comparison of Univariate and Multivariable Odds Ratios
(A) Reference: FFR−/NHPR+……………………………………………………11
(B) Reference: FFR−/NHPR−…………………………………………………...11
Table S5. Summary of Clinical Events……………………………………...…12

Figure S1. Multivariable Generalized Estimating Equation Analysis for the Association Between Physiological Classification Patterns and LRP, with FFR−/NHPR− as the Reference Group

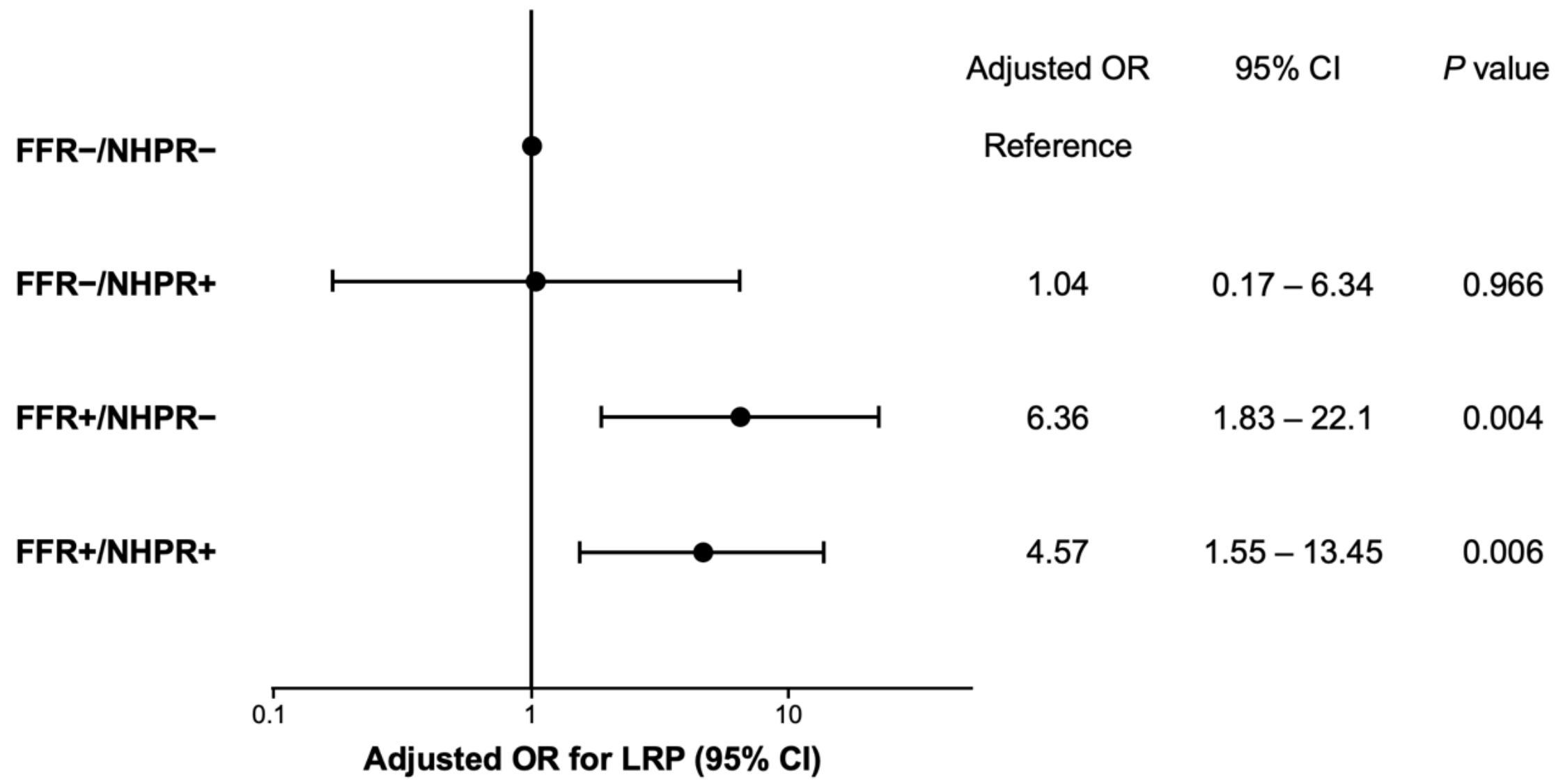

Multivariable generalized estimating equation analysis for the association between physiological classification patterns and the presence of LRP, using FFR−/NHPR− as the reference group. Both the FFR+/NHPR− and FFR+/NHPR+ patterns were independently associated with the presence of LRP, whereas the FFR−/NHPR+ pattern was not.
CI, confidence interval; FFR, fractional flow reserve; NHPR, nonhyperemic pressure ratio; LRP, lipid-rich plaque; OR, odds ratio.

Figure S2. Model Diagnostics for the Multivariable Generalized Estimating Equation Model for the Presence of LRP

(A) QQ plot residuals

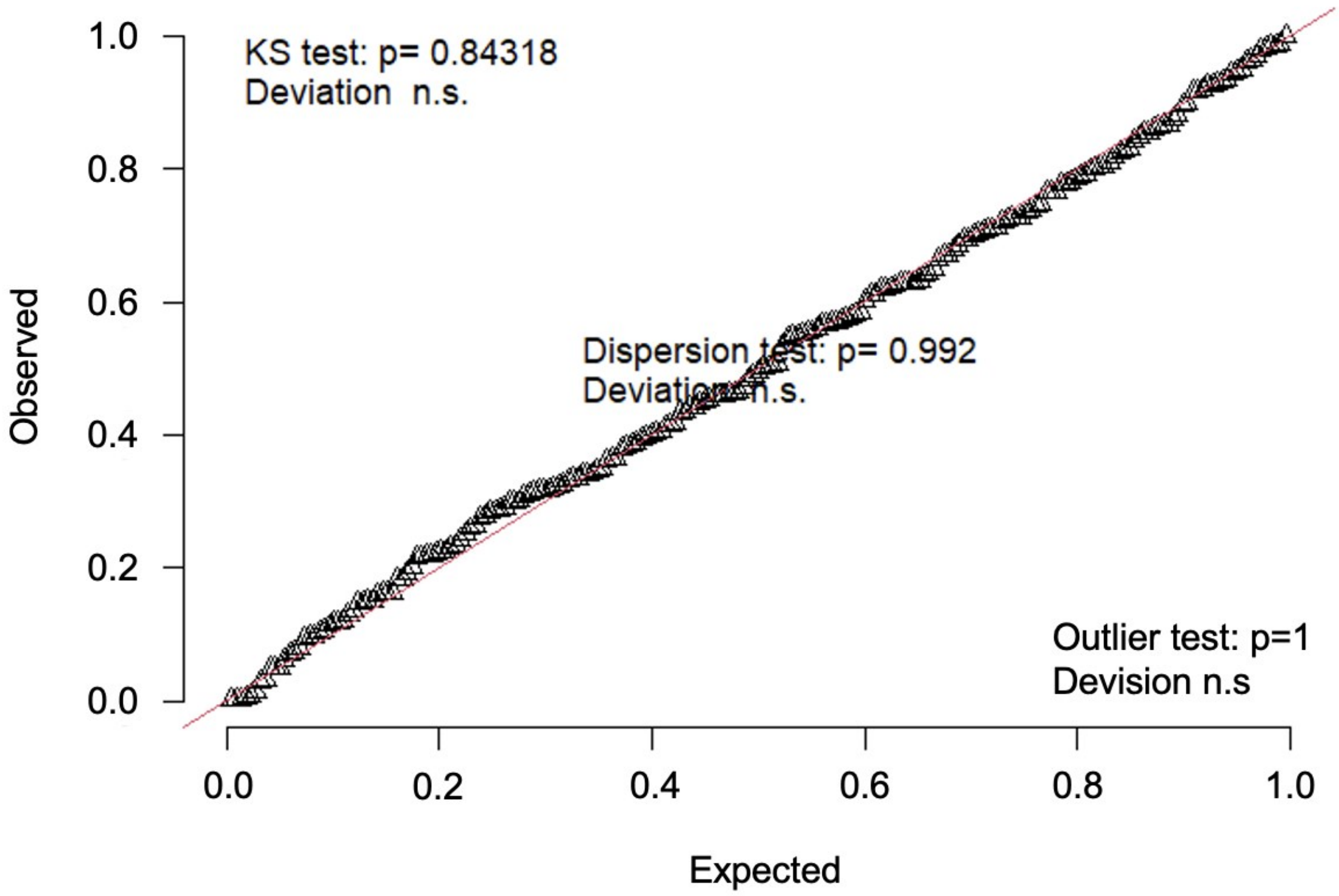

Quantile-quantile (QQ) plot of scaled residuals from the multivariable generalized estimating equation model for the presence of LRP. The observed residual distribution closely followed the expected distribution, with no significant deviation (Kolmogorov–Smirnov test, $P = 0.843$), no evidence of over- or underdispersion (dispersion test, $P = 0.992$), and no significant outliers (outlier test, $P = 1.00$). Abbreviations are as in Figure S1.

(B) DHARMa residual vs predicted

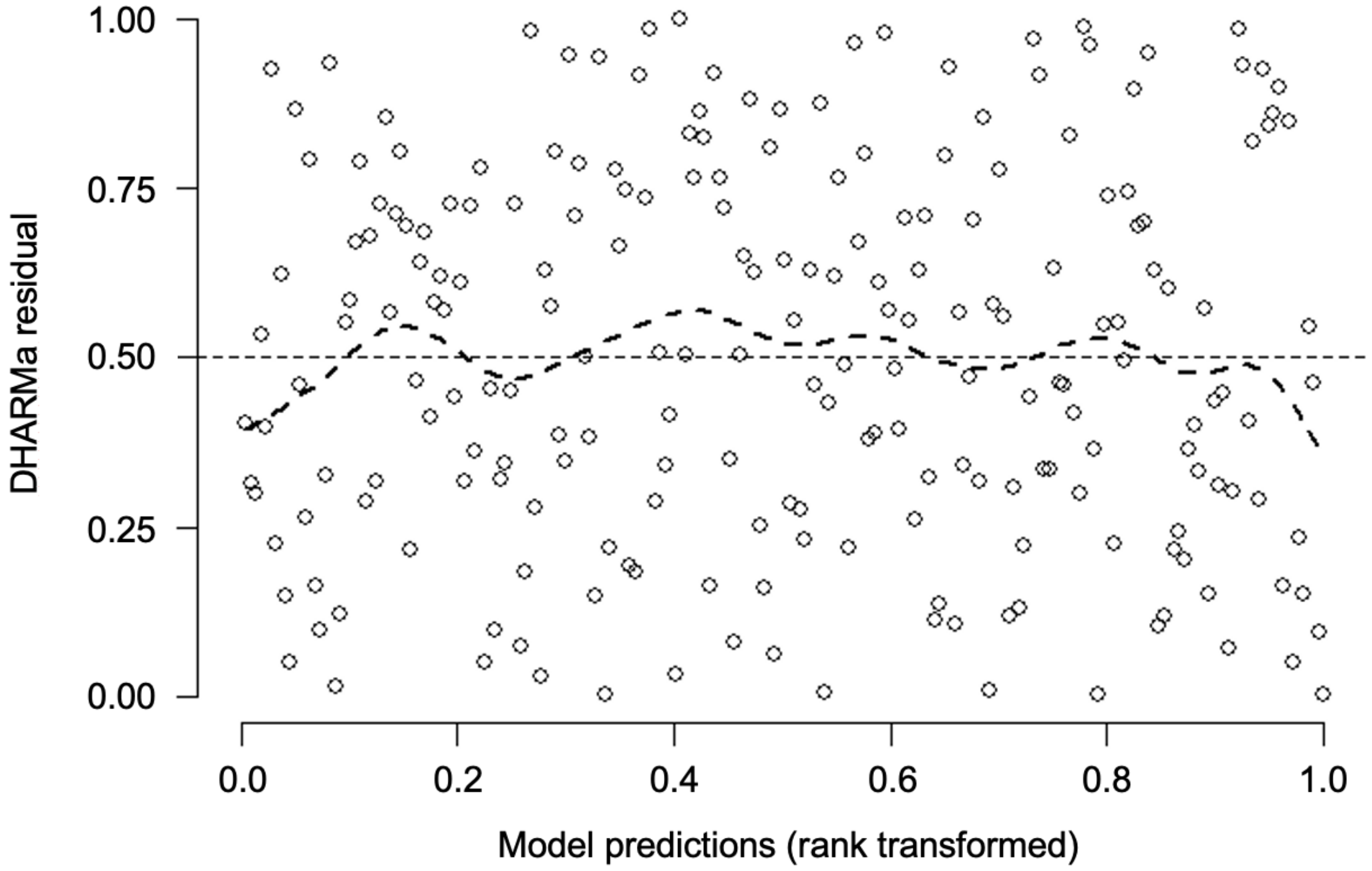

DHARMa residual-versus-predicted plot (rank-transformed model predictions). Residuals were evenly distributed around 0.50 across the range of predicted values, with no systematic pattern indicative of model misspecification. Together, these diagnostic findings did not indicate substantial model misspecification.

Figure S3. Cumulative incidence of TVF across the physiological classification group.

(A) All lesions

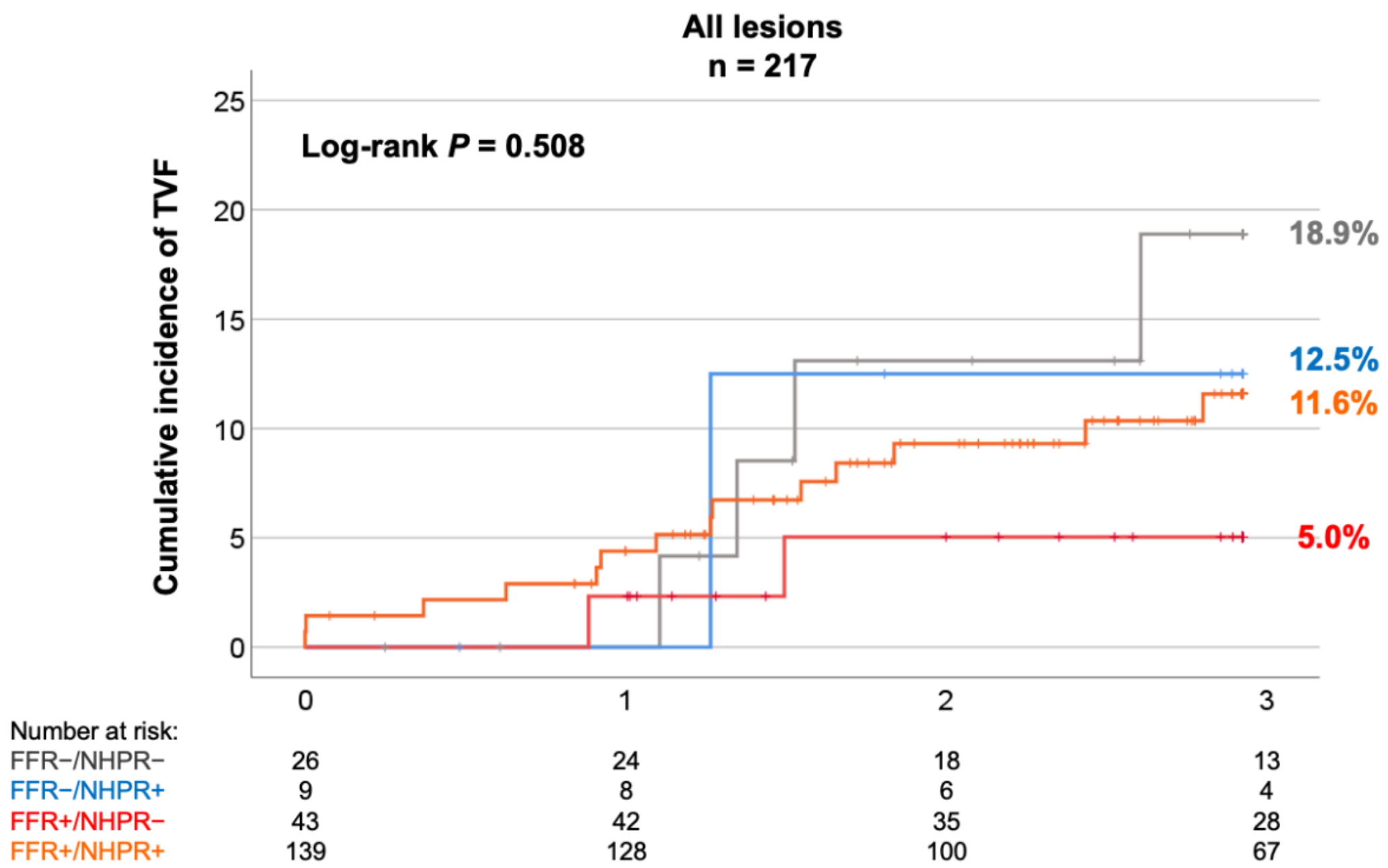

(B) Deferred lesions

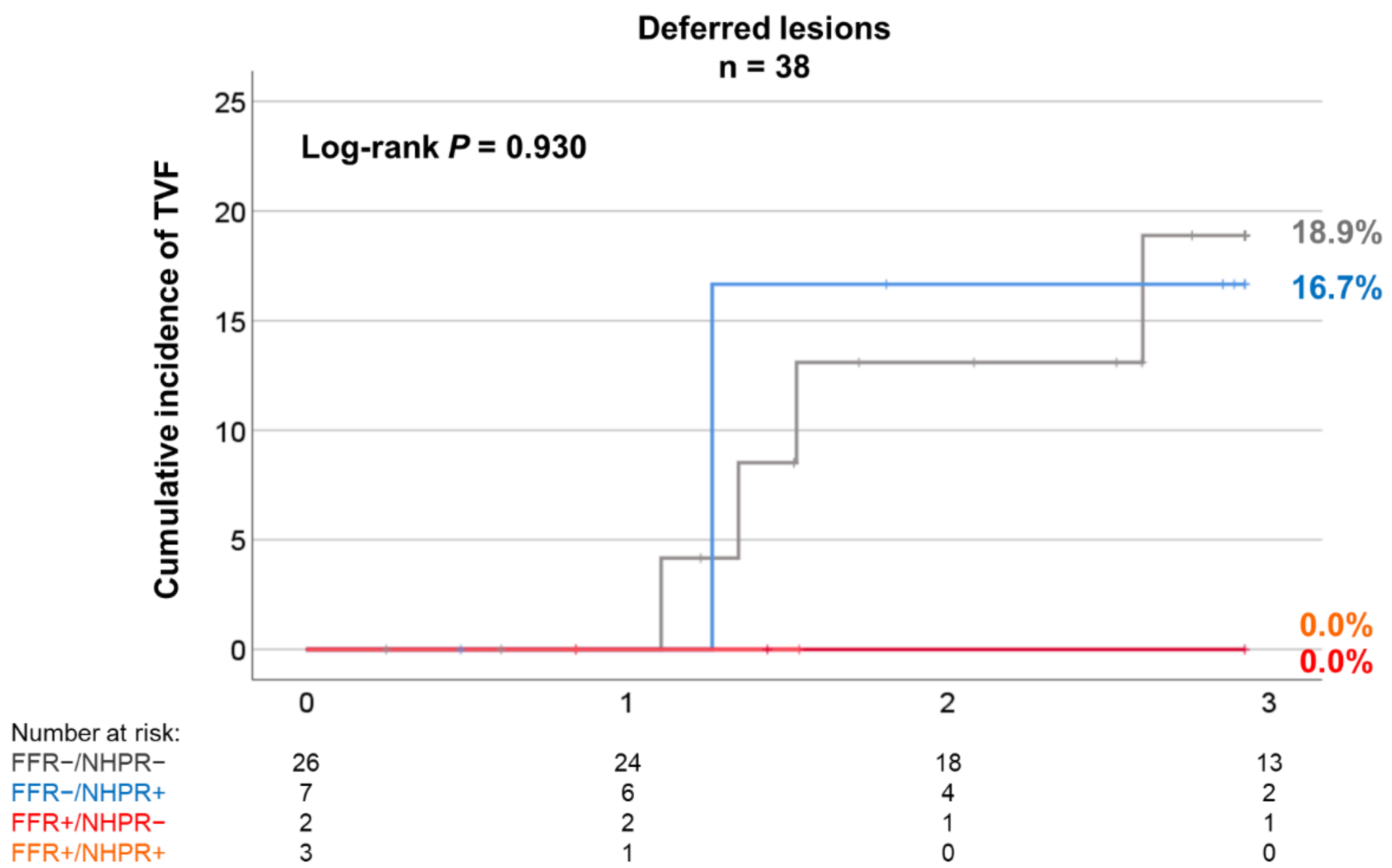

(C) Revascularized lesions

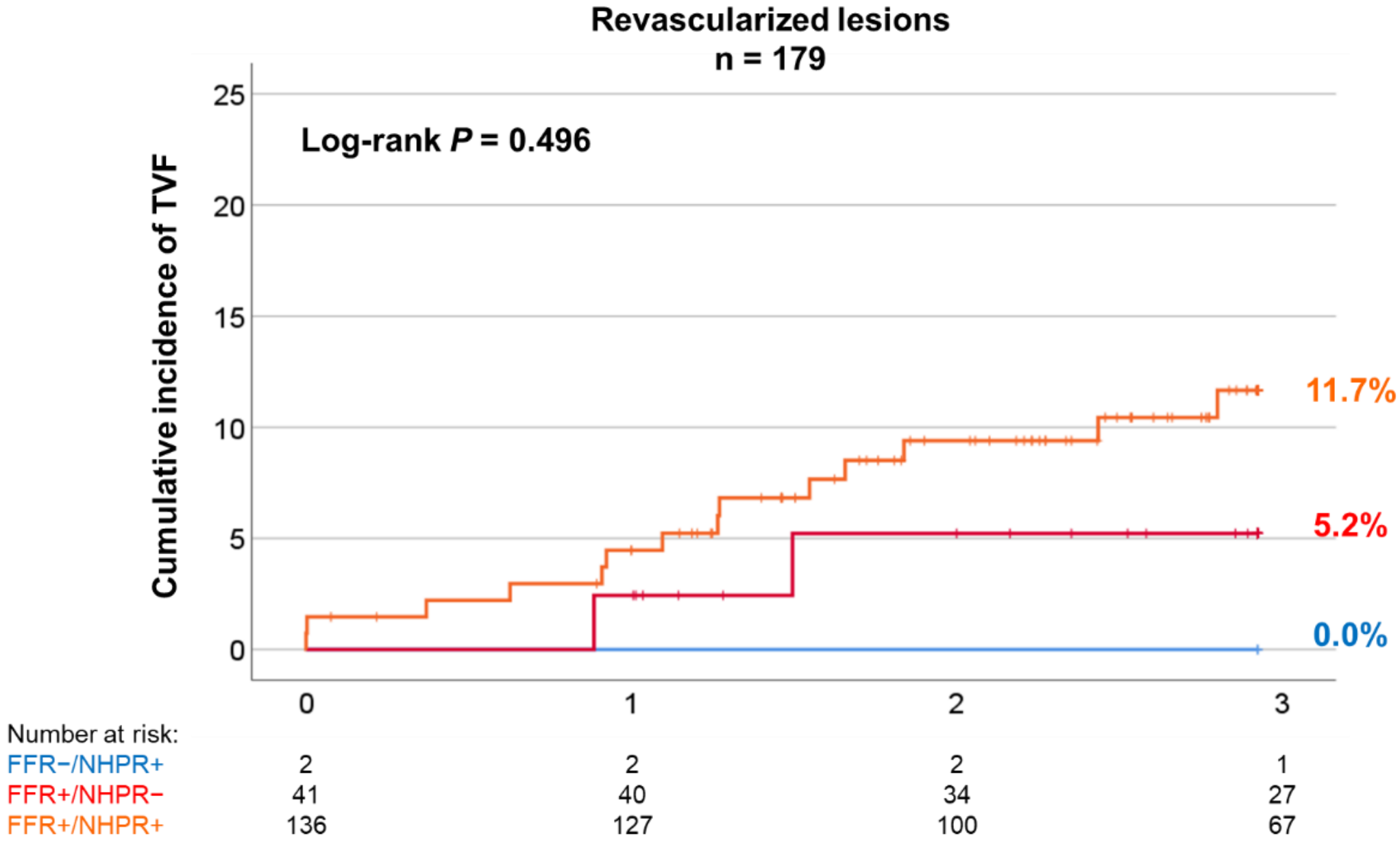

There was no significant difference in the cumulative incidence of TVF among the four physiological classification groups. In subgroup analyses by treatment strategy, the cumulative incidence of TVF did not differ significantly among the four groups for deferred or revascularized lesions.

TVF, target vessel failure; other abbreviations are as in Figure S1.

Table S1. Comparison of Baseline Clinical Characteristics Between Patients Included and Excluded from the Study.

	Include n = 189	Exclude n = 2567	*P* value
Age, years	69.5 ± 10.0	72.8 ± 10.1	<0.001
Male, n (%)	154 (81.5%)	1927 (75.1%)	0.048
Body mass index, kg/m^2	24.6 ± 3.2	24.0 ±3.7	0.032
Acute coronary syndrome	0	404 (15.7%)	-
Hypertension, n (%)	158 (83.6%)	1981 (77.2%)	0.041
Dyslipidemia, n (%)	171 (90.5%)	1970 (76.7%)	<0.001
Diabetes mellitus, n (%)	100 (52.9%)	963 (37.5%)	<0.001
Current smoking, n (%)	25 (13.2%)	261 (10.2%)	0.183
Chronic kidney disease	91 (48.1%)	858 (33.4%)	<0.001
Hemodialysis, n (%)	12 (6.3%)	139 (5.4%)	0.586
Prior myocardial infarction, n (%)	58 (30.7%)	610 (23.8%)	0.032
Prior stroke, n (%)	27 (14.3%)	315 (12.3%)	0.418
Peripheral artery disease, n (%)	25 (13.2%)	311 (12.1%)	0.652

Table S2. Association Between Physiological Classification Pattern and $maxLCBI_{4mm}$ and LRP, Stratified by NHPR Modality

RFR (n = 62)

Characteristic	FFR−/NHPR− n = 7	FFR−/NHPR+ n = 3	FFR+/NHPR− n = 13	FFR+/NHPR+ n = 39	*P* value
$maxLCBI_{4mm}$	356.1 ± 287.4	324.0 ± 99.2	619.3 ± 193.1	427.0 ± 202.1	0.014
LRP, n (%)	2 (29%)	0 (0%)	11 (85%)	21 (54%)	0.016

dPR (n = 146)

Characteristic	FFR−/NHPR− n = 18	FFR−/NHPR+ n = 6	FFR+/NHPR− n = 29	FFR+/NHPR+ n = 93	*P* value
$maxLCBI_{4mm}$	266.7 ± 183.8	379.7 ± 247.2	480.0 ± 195.4	451.2 ± 198.3	0.002
LRP, n (%)	3 (17%)	2 (33%)	16 (55%)	54 (58%)	0.010

DFR (n = 9)

Characteristic	FFR−/NHPR− n = 1	FFR−/NHPR+ n = 0	FFR+/NHPR− n = 1	FFR+/NHPR+ n = 7	*P* value
$maxLCBI_{4mm}$	66.0	-	607.0	608.3 ± 178.4	-
LRP, n (%)	0 (0%)	-	1 (100%)	6 (86%)	-

FFR, fractional flow reserve; LRP, lipid-rich plaque; maxLCBI4mm, maximum lipid core burden index within any 4-mm segment; NHPR, nonhyperemic pressure ratio; RFR, resting full-cycle ratio; dPR, diastolic pressure ratio; DFR, diastolic hyperemia-free ratio.

Table S3. VIF for the Multivariable Model of LRP

Covariate	FFR−/NHPR− reference VIF	FFR−/NHPR+ reference VIF
FFR−/NHPR−	(Reference)	3.06
FFR−/NHPR+	1.47	(Reference)
FFR+/NHPR+	3.18	6.21
FFR+/NHPR−	2.83	4.85
Age	1.17	1.17
Male	1.13	1.13
Diabetes mellitus	1.16	1.16
LDL-C	1.15	1.15
Diameter stenosis	1.50	1.50
Plaque burden	1.18	1.18
Lesion length	1.22	1.22
Lesion location in the LAD	1.12	1.12

LAD, left anterior descending artery; LDL-C, low-density lipoprotein cholesterol; VIF, variance inflation factors; other abbreviations are as in Table S2.

Table S4. Comparison of Univariate and Multivariable Odds Ratios

(A) Reference: FFR−/NHPR+

Physiological pattern	Univariate OR (95% CI)	Multivariable OR (95% CI)
FFR−/NHPR−	0.83 (0.13–5.30)	0.96 (0.16–5.84)
FFR−/NHPR+	(Reference)	(Reference)
FFR+/NHPR+	3.46 (0.72–16.66)	4.39 (0.97–19.84)
FFR+/NHPR−	6.53 (1.20–35.46)	6.12 (1.20–31.28)

(B) Reference: FFR−/NHPR−

Physiological pattern	Univariate OR (95% CI)	Multivariable OR (95% CI)
FFR−/NHPR−	(Reference)	(Reference)
FFR−/NHPR+	1.20 (0.19–7.63)	1.04 (0.17–6.32)
FFR+/NHPR+	3.85 (1.43–10.39)	4.57 (1.55–13.45)
FFR+/NHPR−	7.84 (2.46–25.01)	6.36 (1.83–22.15)

CI, confidence interval; OR, odds ratio; other abbreviations are as in Table S2.

Table S5. Summary of clinical events

	Overall<br>n =217
TVF, n (%)	21 (11.1%)
Cardiac death, n (%)	4 (2.3%)
Target vessel-related non-fatal myocardial infarction, n (%)	6 (2.8%)
Ischemia-driven target vessel revascularization, n (%)	17 (8.9%)

Each event occurrence is based on the Kaplan-Meier estimate at 3 years.
TVF, target vessel failure
